# Isolated laryngeal sarcoidosis

**DOI:** 10.1002/rcr2.502

**Published:** 2019-11-17

**Authors:** Aslan Ahmadi, Fatemeh Dehghani Firouzabadi, Mohammad Dehghani Firouzabadi, Maryam Roomiani

**Affiliations:** ^1^ ENT and Head and Neck Research Center and Department Five Senses Health Research Institute, Hazrat Rasoul Akram Hospital, Iran University of Medical Sciences Tehran Iran

**Keywords:** Airway obstruction, CO_2_ laser therapy, laryngeal sarcoidosis

## Abstract

To improve diagnosis and treatment of laryngeal sarcoidosis, we present a rare case of upper airway obstruction of unclear aetiology, with life‐threatening complication.The patient was a 19‐year boy who presented with progressive severe dyspnoea for eight months. After extensive diagnostic evaluations with no conclusive diagnosis, biopsy showed non‐caseating granulomatous inflammation consistent with laryngeal sarcoidosis that was treated with a CO_2_ laser attached to a microscope. The laser was also used to resect epiglottitis, false focal cords, and aryepiglottic fold completely due to supraglottic swelling with a thick oedematous epiglottis. A 1.5‐ms pulse‐duration CO_2_ laser attached to a microscope is an effective technique of treating laryngeal sarcoidosis and preventing its hazardous complication. Beneficial effects of this method are not only an immediate improvement of the symptoms, but also this method decreases the need for long‐term medical therapy with its side effects or avoid tracheostomy due to upper airway obstruction.

## Abstract 2

To improve diagnosis and treatment of laryngeal sarcoidosis, we present a rare case of upper airway obstruction of unclear aetiology, with life‐threatening complication in a 19‐year‐old boy.

## Graphical abstract

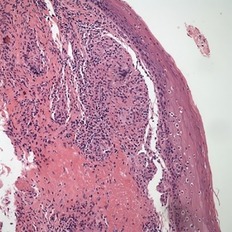

## Introduction

Sarcoidosis is a chronic systemic granulomatosis disease that affects usually lungs, eyes, or skin. Upper respiratory tract involvement is less common as laryngeal sarcoidosis accounts for less than 5% of all patients 1, 2, 3. The major common site of larynx that is affected is supraglottic area such as epiglottis and arytenoids and aryepiglottic folds 4. Occurrence of this disease in young people without lung involvement is rare. Although topical treatment is enough to treat some upper respiratory tract sarcoidosis, systemic treatment is required for most of laryngeal cases. If airway obstruction is evident, excision with carbon dioxide laser or microdebrider can prevent the need for a tracheostomy 5.

## Case Report

**Figure 1:**
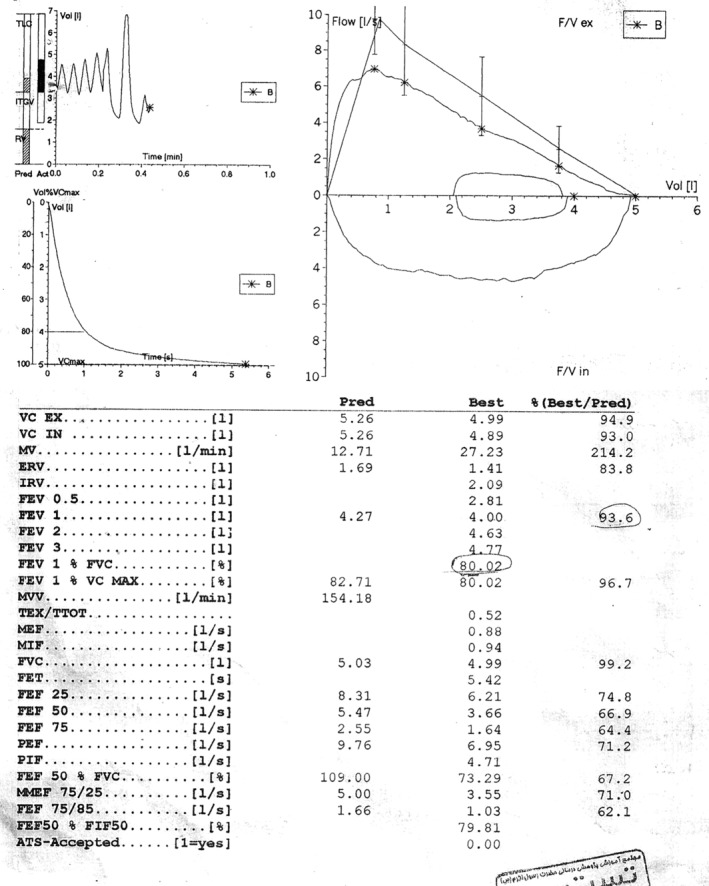
Spirometry results consistent with upper airway obstruction.

**Figure 2:**
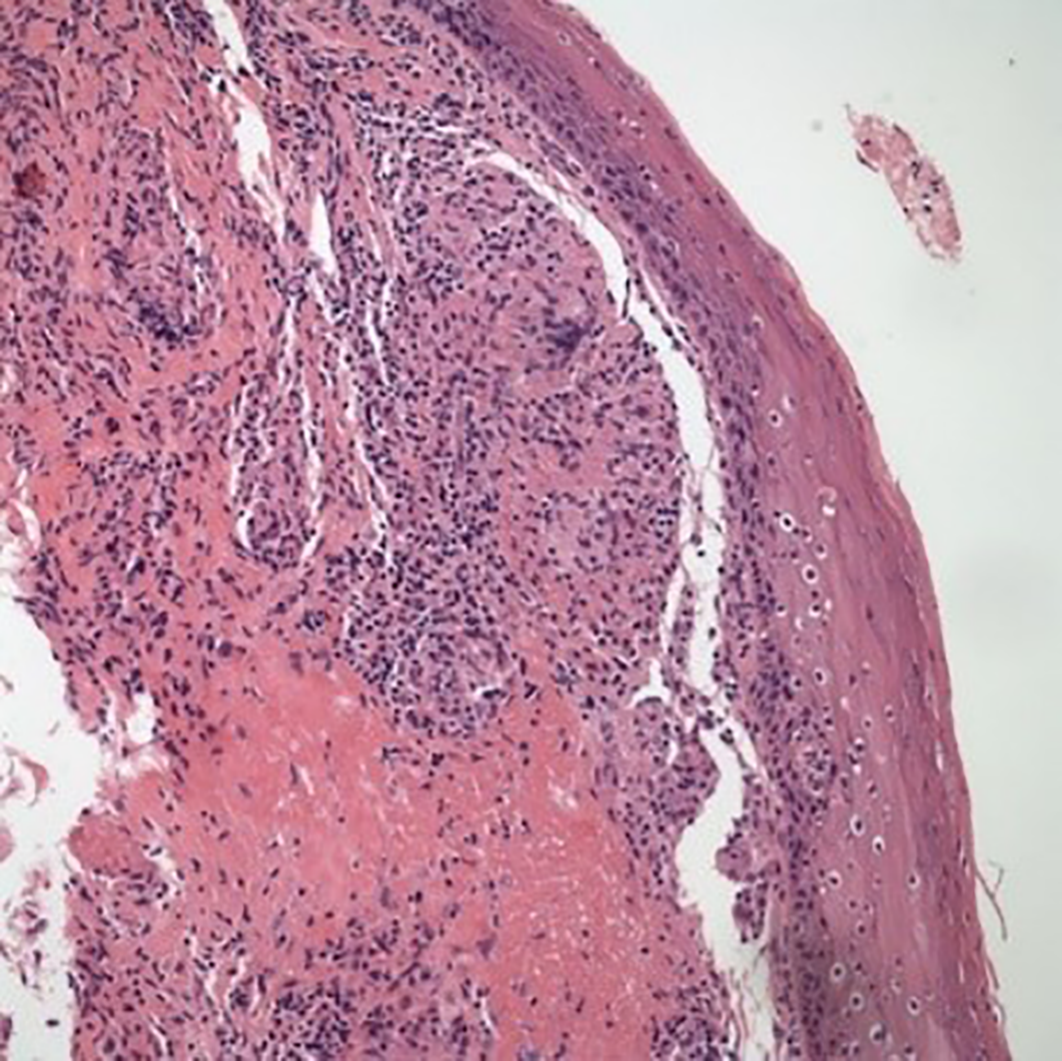
Non‐caseating granulomatous inflammation.

**Figure 3:**
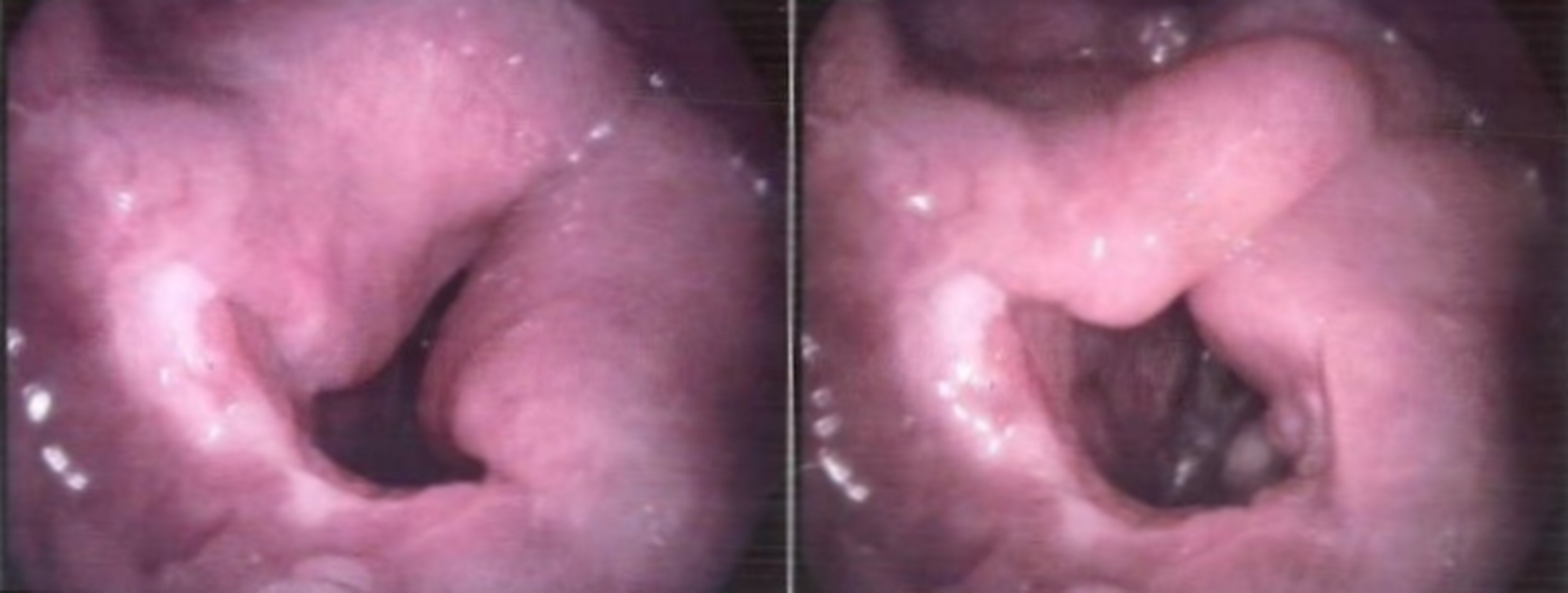
Improvement of laryngeal oedema after CO_2_ laser therapy.

A 19‐year‐old with no past medical history, presented with eight months of progressively worsening snoring and dyspnoea. At initial evaluation, spirometry showed upper airway obstructive pattern (Fig. 1); however, results of other tests such as initial blood testing (white blood cells count, erythrocyte sedimentation rate, C‐reactive protein), rheumatologic tests such as Wagner granulomatosis, and infectious diseases such as tuberculosis were normal based on the direct laryngoscopy and the pathology report. His chest X‐ray was also normal. Treatment with continuous positive airway pressure showed no improvement in patient's symptoms. Direct laryngoscopy was performed and showed turban‐like epiglottitis, oedema of false vocal cords (FVC) and mild inflamed mucosa of trachea. At first, tracheostomy was performed in another centre due to massive supraglotitis oedema and severe dyspnoea. A rheumatologist ordered a biopsy of FVC and epiglottis. The biopsy showed fibro connective tissue and respiratory type mucosa with non‐caseation granulomatous inflammation with marked parenchyma congestion and oedema of FVC and epiglottis that was consistent with sarcoidosis (Fig. 2). Finally, after consulting with the rheumatologist, he went to operating room and after general anaesthesia, supraglutectomy with carbon dioxide laser was done when he did not response to systemic treatment of sarcoidosis. A 1.5‐ms pulse‐duration CO_2_ laser attached to a microscope was used (laser settings of 8 watts, depth of 1.5). The laser was also used to resect epiglottitis, false focal cords and aryepiglottic fold completely due to supraglottic swelling with a thick oedematous epiglottis. Patient was discharged on oral medical therapy after surgery with cefixime and omeprazole and was followed up to 4 weeks. After his final treatment there was resolution of the symptoms and the laryngeal oedema had diminished (Fig. 3). He is currently doing well and has returned to all previous activities without shortness of breath. At four‐week and 12‐week follow‐up visits, he had no respiratory issue and his spirometry and polysomnography were normal, so his tracheostomy tube was removed (after four weeks). Follow‐up laryngoscopies determined considerable recovery in the oedema in the arytenoids and aryepiglottic folds.

## Discussion

Sarcoidosis is a multisystem disease with unknown aetiology. Laryngeal sarcoidosis is a less common that usually was appeared in third decades of patients' life 4. Upper airway obstruction is one of infrequent but serious complication of laryngeal sarcoidosis that occurs in nearly 15% of cases with delays in diagnosis that causes the need for tracheostomy 6. Previous studies have shown the pathognomonic exploration on laryngoscope consists of thickening and enlarged epiglottis that was named as turban‐like epiglottitis, as well as, non‐caseating granuloma on biopsy accounts for landmark of sarcoidosis 7. Treatment of laryngeal manifestations of sarcoidosis can be achieved with systemic corticosteroids. For small ones and well margined, intraregional injection of corticosteroids can be performed. When airway obstruction is apparent carbon dioxide laser or microdebrider is a better choice to avoid the need for tracheostomy. It has shown that resection of lesion or carbon dioxide laser was effective in some cases 5.

Timely diagnosis and treatment of laryngeal sarcoidosis due to obstruction of upper airway that is its hazardous complication is an important issue.

### Disclosure Statement

Appropriate written informed consent was obtained for publication of this case report and accompanying images.
